# A Stereovision Matching Strategy for Images Captured with Fish-Eye Lenses in Forest Environments

**DOI:** 10.3390/s110201756

**Published:** 2011-01-31

**Authors:** Pedro Javier Herrera, Gonzalo Pajares, María Guijarro, José J. Ruz, Jesús M. Cruz

**Affiliations:** 1 Department of Computer Architecture and Automatic Control, Faculty of Computer Science, Complutense University, 28040 Madrid, Spain; E-Mails: jjruz@dacya.ucm.es (J.J.R.); jmcruz@dacya.ucm.es (J.M.C.); 2 Department of Software Engineering and Artificial Intelligence, Faculty of Computer Science, Complutense University, 28040 Madrid, Spain; E-Mails: pajares@fdi.ucm.es (G.P.); mguijarro@fdi.ucm.es (M.G.)

**Keywords:** fish-eye stereovision matching, fuzzy clustering, Bayesian classifier, weighted fuzzy similarity, Hopfield neural networks, texture classification, fish-eye lenses, hemispherical forest images

## Abstract

We present a novel strategy for computing disparity maps from hemispherical stereo images obtained with fish-eye lenses in forest environments. At a first segmentation stage, the method identifies textures of interest to be either matched or discarded. This is achieved by applying a pattern recognition strategy based on the combination of two classifiers: Fuzzy Clustering and Bayesian. At a second stage, a stereovision matching process is performed based on the application of four stereovision matching constraints: epipolar, similarity, uniqueness and smoothness. The epipolar constraint guides the process. The similarity and uniqueness are mapped through a decision making strategy based on a weighted fuzzy similarity approach, obtaining a disparity map. This map is later filtered through the Hopfield Neural Network framework by considering the smoothness constraint. The combination of the segmentation and stereovision matching approaches makes the main contribution. The method is compared against the usage of simple features and combined similarity matching strategies.

## 1. Introduction

### 1.1. Problem Description

One important task in forest analysis is to determine the volume of wood in an area for different purposes, such as to control the degree of growth of the trees or to determine the resources that must be applied for maintenance. The increasing computer vision technologies are demanding solutions for making the above task automatic. One of such technologies is concerned with a stereovision system patented by the Spanish Research Centre (CIFOR) with number MU-200501738. This device, located during the image acquisition at a known 3D position in an identifiable geographical direction, allows us to acquire two stereoscopic hemispherical images with parallel optical axes.

Because of the large areas to be processed in forest environments, a system based on fish-eye lenses allows imaging a large sector of the surrounding space with hemispherical vision. This is the reason by which these systems are suitable for the proposed task. Fish eye optics systems can recover 3D information in a large field-of-view around the camera; in our system it is 183° × 360°. This is an important advantage because it allows one to image the trees in the 3D scene close to the system from the base to the top, unlike in systems equipped with conventional lenses where close objects are partially mapped [1]. Because the trees appear completely imaged, the stereoscopic system allows the calculation of distances from the device to significant points into the trees in the 3D scene, including diameters along the stem, heights and crown dimensions to be measured, as well as determining the position of the trees. These data may be used to obtain precise taper equations, leaf area or volume estimations [2]. As the distance from the device to each tree can be calculated, the density of trees within a determined area can be also surveyed and growing stock; tree density, basal area (the section of stems at 1.30 m height in a hectare) and other interesting variables may be estimated at forest stand level using statistical inference [3].

Moreover, the images constitute a permanent record of the sample point that allows measurement error control and future data mining, which currently requires revisiting the plot. Currently, the above mentioned measurements are obtained manually. An important goal is the automation of the process for data acquisition. Hence, a passive stereovision-based system is a suitable technique for this task, because during the intervention the trees are not affected by the measurement.

According to [4,5], we can view the classical problem of stereo analysis as consisting of the following steps: image acquisition, camera modelling, feature extraction, image matching and depth determination. The key step is that of image matching. This is the process of identifying the corresponding points in two images that are cast by the same physical point in the 3-D space. This paper is devoted to the feature extraction and image matching steps.

**Figure 1.**
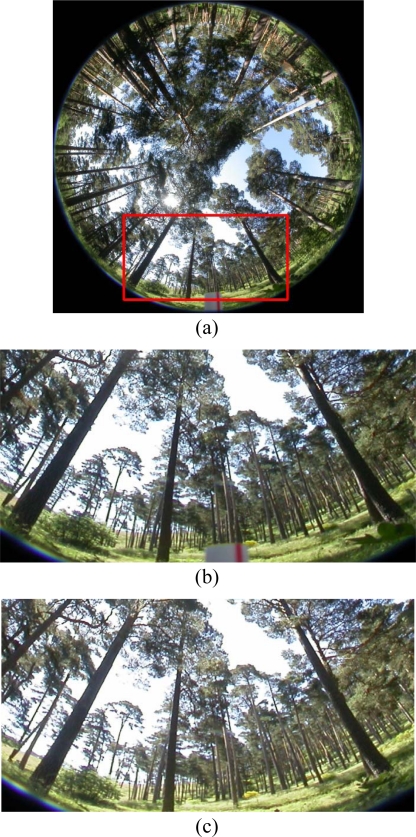
**(a)** Hemispherical left image; **(b)** left expanded area; **(c)** corresponding right expanded area.

In our approach, the interest is focused on the trunks of the trees because they contain the higher concentration of wood. These are our features of interest in which the matching process is focused. Figure 1(a) displays a representative hemispherical image of the stereo pair (let’s say the left one) captured with a fish-eye lens of the forest. As one can see there are three main groups of textures out of interest, such as grass in the soil, sky in the gaps and leaves of the trees. Hence, the first step consists on the identification of the textures out the interest to be excluded during the matching process. This is carried out through a segmentation process which uses both: (a) methods for texture analysis [6] and (b) a classification approach based on the combination of two single classifiers, they are the well-known fuzzy clustering strategy [7] and the parametric Bayesian estimator [8].

The first tries to isolate the leaves based on statistical measures and the second classifies the other two kinds of textures. The performance of combined classifiers has been reported as a promising approach against individual classifiers [9]. One might wonder why not to identify the textures belonging to the trunks. The response is simple. This kind of textures displays a high variability of tonalities depending on the orientation of the trunks with respect the sun, as detailed later in Section 2. Therefore, there is not a unique type of texture (dark or illuminated trunks and even though alternatively in bits), as we can see in Figure 1(a).

Once the textures to be excluded have been identified, now the goal is to match trunks between the two images of the stereo pair. Figure 1(b) displays the signed and expanded area on Figure 1(a). This is intended for making more explicit the details. In Figure 1(c) the corresponding area in the right image of the stereo pair is displayed.

Because of the irregular forms and distribution of the trunks, the most suitable features to be matched are pixels. For such a purpose we exclude the pixels identified as belonging to one of the three kinds of textures out of interest mentioned above. The remaining pixels are the candidates to be matched, where those belonging to the trunks must be found.

Moreover, as the images are captured in two positions separated a certain distance (base-line), the tree’s crowns are located at different positions with respect each camera position and the incident rays of the sun produce important lighting variability between the pixels locations and surrounding areas in both images for the same structure in the scene; this makes the matching process a difficult task. This observation is applicable for the whole images.

In stereovision matching there are a set of constraints that are generally applied for solving the matching problem, such as: epipolar, similarity, uniqueness or smoothness.

*Epipolar*: derived from the system geometry, given a pixel in one image its correspondence in the other image will be on the unique line where the 3D spatial points belonging to a special line are imaged. *Similarity*: matched pixels have similar attributes or properties. *Uniqueness*: a pixel in the left image must be matched to a unique pixel in the right one, except for occlusions. *Smoothness*: disparity values in a given neighbourhood change smoothly, except at a few discontinuities belonging to the edges, mainly in the trunks.

Two sorts of techniques have also been broadly used for matching [5]: area-based and feature based. Area-based stereo techniques involve brightness (intensity) patterns in the local neighbourhood of a pixel in one image and the brightness patterns in the local neighbourhood of the corresponding pixel in the other image. Two kinds of approaches fall into this category. The first is concerned with the correlation coefficient and the second with statistical measures, generally used for identifying textures. Feature-based methods [10] compute some attributes for the pixels under correspondence; they can be simple attributes, such as the colour of the pixels or properties obtained by applying some operator such as the gradient (module and direction) or Laplacian. They were satisfactorily used in [11], although some of them, such as the Laplacian, could become noise sensitive in some contexts. Really, these operators take into account the pixels and its neighbours; hence, from this point of view they could be considered as area-based. The colour is the unique attribute where the neighbourhood is not involved.

### 1.2. Motivational Research

The correspondence process is designed as follows. Given a pixel in the left image, we apply the epipolar constraint for determining a list of candidates, which are potential matches, in the right image. Each candidate becomes an alternative for the pixel in the left image. For each pair of pixels, we apply the similarity constraint based on the six attributes mentioned above: (a) correlation coefficient, (b) variance as a measure of the texture, (c) colour for each pixel, (d) gradient magnitude, (e) gradient angle and (f) Laplacian. The gradient is computed through the Sobel operator, although some other edge operators could be used. Based on the six attributes we compute six similarity measures, between a pixel and the pixels in the list of candidates. These similarity measures are conveniently combined. The final decision about the correct match, among the candidates in the list, is made according to the support that each candidate receives by applying a decision making strategy based on a Weighted Fuzzy Similarity (WFS) approach. The unique selection made about the correct match implies the application of the uniqueness constraint. In summary, at this moment we have applied three stereovision matching constraints (epipolar, similarity and uniqueness) and a disparity map is built taking as reference each pixel location in the left image. The disparity value at this location is the absolute difference value in sexagesimal degrees between the angle for the pixel in the left image and the angle of its matched pixel in the right one. Each pixel is given in polar coordinates with respect the centre of the image. This is detailed in Section 3.3.

Now the goal is to improve the disparity map up to where it is possible. Erroneous disparity values must be removed and the disparities associated to pixels belonging to the trunks must be smoothed. These two sub-goals, can be achieved by applying the stereovision smoothness constraint, where it considers not only the isolated disparity values at each pixel location but the pixels in the neighbourhood. For such purpose we have selected the Hopfield Neural Network (HNN) paradigm because it can cope with this. Indeed, it is an optimization approach, which can be controlled by energy minimization, making it a suitable approach. Moreover, the HNN has been used satisfactorily in stereovision vision matching approaches although in a different context and under different criteria [12].

### 1.3. Contribution and Organization of This Paper

The images analyzed belong to Scots pine (*Pinus sylvestris* L.) forests; Figure 1 displays a representative image. This paper presents the combination of a segmentation process for identifying three kinds of textures and a stereovision matching process, where the WFS approach allows the mapping of the similarity and uniqueness constraints obtaining an initial disparity map. This map is later filtered for its improvement by applying the smoothness stereovision matching constraint through the HNN paradigm. The proposed approach is compared favourably against the usage of individual area-based and feature-based matching techniques and against other combined decision making approaches.

This work is organized as follows. In Section 2 we describe the procedures applied for the image segmentation oriented to the identification of textures. Section 3 is split in two parts; the first describes the design of the matching process by applying the epipolar, similarity and uniqueness constraints; including the overview of the WFS approach. The second part describes the HNN paradigm and the method for applying the smoothness constraint. Section 4 displays the results obtained by using the proposed approach, and compares them with those obtained by considering the individual similarities, also by applying only the WFS strategy, and others combined decision making strategies. Section 5 presents the conclusions and future work.

## 2. Image Segmentation

The images analysed belong to different pinewoods, Figure 1 displays a representative image. As mentioned before, the goal of the image segmentation process is to exclude the pixels belonging to one of the three kinds of textures out of interest: sky, grass in the soil and leaves. The exclusion of these textures is useful because the errors that they could introduce during the correspondence can be considerably reduced. This justifies the application of the segmentation process.

Observing the textures we can see the following: (a) the areas occupied with leaves display high intensity variability in a pixel and the surrounding pixels in its neighbourhood; therefore methods based on detecting this behaviour could be suitable; (b) on the contrary, the sky displays homogeneous areas, where a pixel is surrounded with neighbouring pixels with similar intensity values where the dominant spectral visible component is blue; (c) the grass in the soil also tend to fall on the category of homogeneous textures although with some variability coming from shades, in both shading and sunny areas the pixels belonging to the grass have the green spectral component as the dominant one; (d) the textures coming from the trunks are the most difficult; indeed due to the sun position, the angle of the incident rays from the sun produce strong shades in the part of the trunks in the opposite position of the projection [west part in the image of Figure 1(a)]; the trunks receiving the direct projection display a high degree of illumination [east part in the image of Figure 1(a)]; there are a lot of trunks where the shades produce different areas.

Based on the above, the identification of the trunks based on texture analysis is a difficult task. For identifying the textures coming from leaves, we use texture analysis techniques based on statistical measures that can cope with the high intensity variability (Section 2.1). Because of the homogeneity of grass and sky textures we can use methods based on learning approaches as explained in Section 2.2. Finally, only trunk pixels participate in the matching process, described in Section 3.

### 2.1. Identification of High Contrasted Textures

A variety of techniques have been used for texture identification [13]. Most techniques rely on comparing values of what are known as second-order statistics [6]. These methods calculate measures of image texture such as the degree of contrast, coarseness, entropy or regularity; or periodicity, directionality and randomness [14]. Alternative methods of texture analysis for image retrieval include the use of Gabor filters localized in space and frequency, which can be used to retrieve frequential properties of a texture [15]; wavelets which identify the textures based on the image decomposition on different sub-bands according to the orientation [16]; fractals used as measures of complexity for identifying repetitive patterns [17]; Fourier based for computing the orientation and spatial period for textures with at least two prominent directions [18].

The textures produced by the leaves of the trees under analysis do not display spatial distributions of frequencies nor textured patterns; they are rather high contrasted areas without any spatial orientation. Hence, we have verified that the most appropriate texture descriptors are those capturing the high contrast, *i.e.*, statistical second-order moments.

One of the simplest is the variance *σ*^2^ (*z*) [6]. It is a measure of intensity contrast defined according to the *z* intensity levels. An intensity contrast coefficient, normalized in the range [0, +1] can be defined as in [6]:

(1) $$Z=1-\frac{1}{1+\sigma^{2}(z)}$$

As one can see, *Z* is 0 for areas of constant intensity, where the variance is zero, and approaches +1 for large values of *σ*^2^ (*z*), *i.e.*, high contrasted areas. This measurement is taken on the intensity image in the HSI colour model transformed from the original RGB. Only pixels with a value for the coefficient *Z* greater than a threshold *T*_1_, fixed to 0.8 in this paper, are allowed to ensure that only pixels belonging to leaves are excluded, *i.e.*, with high contrast.

### 2.2. Fuzzy Clustering and Bayesian Estimator Combination: Identifying Relevant Smooth Textures

As mentioned before, in our approach there are two relevant textures that must be identified. They are specifically the sky and the grass. A pixel belonging to one of such textures displays a low value for *Z* because of its homogeneity. This is a previous criterion for identifying such areas, *Z* < *T*_1_. Nevertheless, this is not sufficient because there are other different areas which are not sky or grass fulfilling this criterion. Therefore, we apply a classification technique based on the combination of the Fuzzy Clustering (FC) and the parametric Bayesian estimator (PB) approaches. These classifiers are selected because of their individual performances in many classification approaches. According to [9], if they are combined the results improve. Both FC and PB consists of two phases: training and decision.

#### 2.2.1. Training Phase

We start with the observation of a set *X* of *n* training patterns, *i.e.*, *X* = {***x***_1_, ***x***_2_,…,***x****_n_*} ∈ ℜ*^d^*. Each sample is to be assigned to a given cluster *c_j_*, where the number of possible clusters is *c*, *i.e.*, *j* = 1, 2,…, *c*. In our approach the number of clusters is two corresponding to grass and sky textures, *i.e.*, *c* = 2. For simplicity, in this paper, we identify the cluster *c*_1_ with the sky and the cluster *c*_2_ with the grass. The ***x****_i_* patterns represent pixels in the *RGB* colour space. Their components are the *R*,*G*,*B* spectral values. This means, that the dimension of the space ℜ is *d* = 3.

##### (a) Fuzzy Clustering (FC)

This training process receives the input training patterns, which have been previously classified as belonging to one of the above clusters *c*_1_ and *c*_2_. According to [7,8], FC computes for each ***x****_i_* at the iteration *k* its membership grade *μ_ij_* and updates the cluster centres for each cluster, ***v****_j_* ∈ ℜ*^d^* according to Equation (2):

(2) $$\mu_{\text{ij}}\;\left(k+1\right)=\frac{1}{\sum_{r=1}^{c}{\left(d_{\text{ij}}\;\left(k\right)/d_{\text{ir}}\;\left(k\right)\right)}^{2/\left(e-1\right)}};{\text{v}}_{j}\;\left(k+1\right)=\frac{\sum_{i=1}^{n}\mu_{\text{ij}}^{e}\left(k\right){\text{x}}_{i}}{\sum_{i=1}^{n}\mu_{\text{ij}}^{e}\left(k\right)}$$

$d_{\text{ij}}^{2}\equiv d^{2}\left({\text{x}}_{i},{\text{v}}_{j}\right)$ is the squared Euclidean distance. The number *e* is called the exponent weight [7,19] fixed to 2.1 in our experiments. The stopping criterion of the iteration process is achieved when ‖*μ_ij_*(*k* + 1) − *μ_ij_*(*k*)‖ < *ɛ* ∀*ij* or a number *k_max_* of iterations is reached, set to 20 in our experiments; *ɛ* has been set to 0.1 in this paper, both fixed after trial and error.

The method requires the initialization of the cluster centres, so that the Equation (2) can be applied at the iteration *k* = 1. With such purpose we apply the pseudorandom procedure described in [20]:

Perform a linear transform *Y* = *f*(*X*) of the training sample values so that they range in the interval [0,1].

Initialize ***v*** = 2*D****M̄* ○ *R*** + *D****m̄,*** where ***m̄*** is the mean vector for the transformed training samples values in *Y* and ***M̄*** = max(*abs*(*Y* − ***m̄***)), both of size 1 × *d*; *D* = [1 … 1]*^t^* with size *c ×* 1; ***R*** is a *c × d* matrix of random numbers in [0,1]; the operation ○ denotes the element by element multiplication.

##### (b) Parametric Bayesian Estimator (PB)

This estimator assumes a known distribution (generally Gaussian) for each cluster expressed as follows [8]:

(3) $$p\left(\text{x}|c_{j}\right)=\frac{1}{{\left(2\pi \right)}^{d/2}{\left|C_{j}\right|}^{1/2}}\text{exp}\left[-\frac{1}{2}{\left(\text{x}-{\text{v}}_{j}\right)}^{t}C_{j}^{-1}\left(\text{x}-{\text{v}}_{j}\right)\right]$$

where the parameters to be estimated are the mean ***v****_j_* and the covariance *C_j_*, both for each cluster *c_j_*. They are estimated through maximum likelihood as:

(4) $${\text{v}}_{j}=\frac{1}{n_{j}}\sum_{k=1}^{n_{j}}{\text{x}}_{k}\;\;\;C_{j}=\frac{1}{n_{j}-1}\sum_{k=1}^{n_{j}}\left({\text{x}}_{k}-{\text{v}}_{j}\right){\left({\text{x}}_{k}-{\text{v}}_{j}\right)}^{T}$$

where *T* denotes transpose and *n_j_* is the number of samples in the cluster *c_j_*.

#### 2.2.2 Decision Phase

After the training phase, a new unclassified sample ***x****_s_* ∈ ℜ*^d^* must be classified as belonging to a cluster *c_j_*. Here, each sample, like each training sample, represents a pixel at the image with the *R*,*G*,*B* components. FC computes the membership degrees for ***x****_s_* to each cluster according to the Equation (2) and PB computes the probabilities that ***x****_s_* belong to each cluster from the Equation (3). Both, probabilities and membership degrees, are the outputs of the individual classifiers ranging in [0, 1]. They are combined by using the *mean rule m_sj_* = 0.5 (*μ_sj_* + *p*(***x****_s_* | *c_j_*))[9] which outperforms other combined schemes studied in [21], specially in the RGB colour model as reported in [22,23]. The pixel represented by ***x****_s_* is classified according to the following decision rule: ***x****_s_* ∈ *c_j_* if *m_sj_* > *m_sh_* and *m_sj_* > *T*_2_ otherwise the pixel remains unclassified. We have added, to the above rule, the second term with the logical “and” operator involving the threshold *T*_2_ because we are only identifying pixels belonging to the sky or grass clusters. This means that the pixels belonging to textures different from the previous ones remain unclassified, and they becomes candidates for the stereo matching process. The threshold *T*_2_ has been set to 0.8 after experimentation. This is a relative high value, which identifies only pixels with a high membership grade of belonging to either *c*_1_ or *c*_2_. We have preferred to exclude only pixels which belong clearly to one of the above two textures.

**Figure 2.**
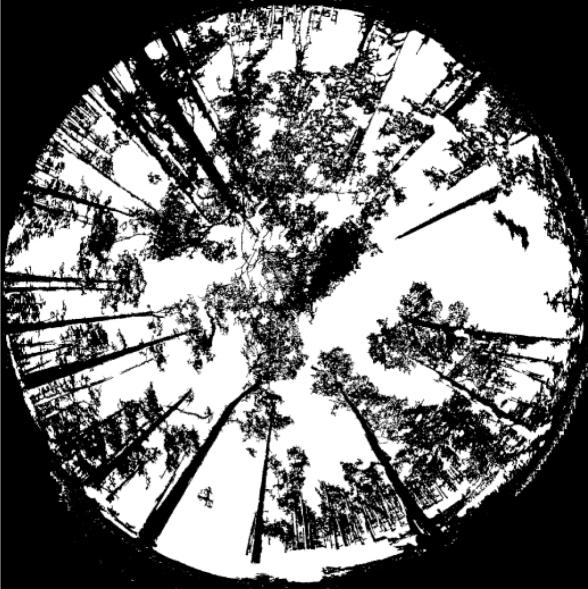
Segmented image, where white areas are textures out of interest (sky, grass and leaves) and the black ones the pixels to be matched.

Figure 2 displays the result of applying the segmentation process to the image in Figure 1. The white areas are identified either as textures belonging to sky and grass or leaves of the trees. On the contrary, the black zones, inside the circle defining the image, are the pixels to be matched. As one can see the majority of the trunks are black, they really represent the pixels of interest to be matched through the corresponding matching process. There are white trunks representing trees very far from the sensor. They are not considered because are out of our interest, as explained in Section 4.

## 3. Stereovision Matching Process

Once the image segmentation process is finished, we have identified pixels belonging to three types of textures which are to be discarded during the next stereovision matching process, because they are out of interest. Hence, we only apply the stereovision matching process to the pixels which remain unclassified.

As mentioned during the introduction, in stereovision there are several constraints that can be applied. In our approach we apply four of them, *i.e.*, epipolar, similarity, uniqueness and smoothness. The epipolar allows restricting the search space for correspondence. The similarity and uniqueness, which are based on the WFS approach allows computing an initial disparity map, which is refined through the HNN approach. The three first ones constraints are addressed in Subsections 3.1 to 3.2. This initial disparity map is described in Section 3.3. Finally, the smoothness constraint, mapped under the HNN is explained in Section 3.4.

### 3.1. Epipolar: System Geometry

**Figure 3.**
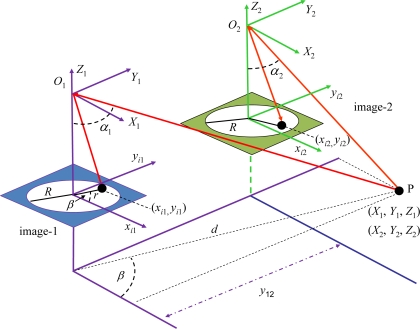
Geometric projections and relations for the fish-eye based stereo vision system.

Figure 3 displays the stereo vision system geometry [1,24]. The 3D object point *P* with world coordinates with respect to the systems (*X*_1_, *Y*_1_, *Z*_1_) and (*X*_2_, *Y*_2_, *Z*_2_) is imaged as (*x*_i1_, *y*_i1_) and (*x*_i2_, *y*_i2_) in image-1 and image-2 respectively in coordinates of the image system; *α*_1_ and *α*_2_ are the angles of incidence of the rays from *P*; *y*_12_ is the baseline measuring the distance between the optical axes in both camera positions (image-1 and image-2) along the *y*-axes; *r* is the distance between image point and optical axis; *R* is the image radius, identical in both images.

According to [25], the following geometrical relations can be established:

(5) $$r=\sqrt{x_{i1}^{2}+y_{i1}^{2}};\;\alpha_{1}=\left(r\pi \right)/R;\;\beta ={\mathrm{tg}}^{-1}\left(y_{i1}/x_{i1}\right)$$

Now the problem is that the 3D world coordinates (*X*_1_, *Y*_1_, *Z*_1_) are unknown. They can be estimated by varying the distance *d* as follows:

(6) $$X_{1}=d\;\text{cos}\beta ;Y_{1}=d\;\text{sin}\beta ;\;\;Z_{1}=\sqrt{X_{1}^{2}+Y_{1}^{2}}/\text{tan}\;\alpha_{1}$$

From Equation (6) we transform the world coordinates in the system *O*_1_*X*_1_*Y*_1_*Z*_1_ to the world coordinates in the system *O*_2_*X*_2_*Y*_2_*Z*_2_ taking into account the baseline as follows:

(7) $$X_{2}=X_{1};\;Y_{2}=Y_{1}+y_{12};\;Z_{2}=Z_{1}$$

Assuming no lenses radial distortion, we can find the imaged coordinates of the 3D point in image-2 as [25]:

(8) $$x_{i2}=\frac{2R\;\text{arctan}\left(\sqrt{X_{2}^{2}+Y_{2}^{2}}/Z_{2}\right)}{\pi \sqrt{{\left(Y_{2}/X_{2}\right)}^{2}+1}},\;y_{i2}=\frac{2R\;\text{arctan}\left(\sqrt{X_{2}^{2}+Y_{2}^{2}}/Z_{2}\right)}{\pi \sqrt{{\left(X_{2}/Y_{2}\right)}^{2}+1}}$$,

Using only a camera or a camera position, we capture a unique image and the 3D points belonging to the line $\bar{O_{1}P}$, are all imaged on the unique point represented as (*x*_*i*1_, *y*_*i*1_). So, the 3D coordinates with a unique image cannot be obtained. When we try to match the imaged point (*x*_*i*1_, *y*_*i*1_) into the image-2 we follow the epipolar line, *i.e.*, the projection of $\bar{O_{1}P}$ over the image-2. This is equivalent to vary the parameter *d* in the 3-D space. So, given the imaged point (*x*_*i*1_, *y*_*i*1_) in the image-1 (left) and following the epipolar line, we obtain a list of *m* potential corresponding candidates represented by (*x*_*i*2_, *y*_*i*2_) in the image-2 (right).

### 3.2. Similarity and Uniqueness: Based on the WFS Approach

Each pixel *l* in the left image is characterized by its set of attributes *A_l_* = (*A_la_*, *A_lb_*, *A_lc_*, *A_ld_*, *A_le_*, *A_lf_*) where the *A_lj_* are identified with the six properties computed for each pixel, *i.e.*, the sub-index *j* = *a*,*b*,*c,d*,*e*,*f*. In the same way, each candidate *i* in the list of *m* candidates is described by its set of attributes *A_i_*, such that *A_i_* = (*A_ia_*, *A_ib_*, *A_ic_*, *A_id_*, *A_ie_*, *A_if_*). The weights associated with every attribute are respectively *w* = (*w_a_*, *w_b_*, *w_c_*, *w_d_*, *w_e_*, *w_f_*), estimated according to its relative importance or relevance, as described later in the Section 4.2. We have verified during our experiments that the attributes used for matching display high variability in their values. Indeed, the differences between attributes for true matches sometimes become greater than differences between false matches. This leads to a high degree of uncertainty or imprecision when a decision about the correct match is to be made. The fuzzy set theoretic techniques provide a general framework to deal with imprecision. This is the main reason for applying the similarity stereovision matching constraint under the fuzzy set theory paradigm. In [26] is described three similarity measures defined in [27], all they display a similar behaviour in our approach and therefore we have chosen the one defined in Equation (9) because of its lower computational cost. For this purpose the attributes *A_lj_* and *A_ij_* are linearly mapped to range in the interval [0, 1]. The lower and upper limits for the six attributes used for normalization are: (a) correlation [−1, 1], which are the usual values; (b) colour [0, 765] corresponding to equal values (zero) for both attributes or opposite (*i.e.*, three zeros and the other three 255); (c) texture [0, 85], computed as the standard deviation in a 3 × 3 neighbourhood, *i.e.*, the lower is zero if all values are equal and the upper 85 if a value is zero and the remainder 255 or vice versa; (d) gradient magnitude [0, 255] minimum and maximum difference between values; (e) gradient direction [0, 360°] around the circle; (f) Laplacian [0, 2,040], where if all values in the neighbourhood are equal, the Laplacian is zero and if the central pixel is zero and the remainder 255 or vice versa, the Laplacian value is 8 × 255.

Once these values are normalized to such range they can be considered as fuzzy measures. From the point of view of the fuzzy theory, the sets *A_l_* and *A_i_* are considered as fuzzy ones and their attributes *A_lj_* and *A_ij_* as membership degrees. Given the pixels *l* and *i*, under the above consideration we can measure its similarity as follows:

(9) $$d_{i}\;(A_{l},\;A_{i})=\frac{1}{\text{card}\;\left(A_{l}\right)}\sum_{j}\left(1-w_{j}\left|A_{\text{lj}}-A_{\text{ij}}\right|\right),\;j=a,b,c,d,e,f$$

where *card* (*A_l_*) denotes the cardinal of the set *A_l_* or equivalently the cardinal of the set *A_i_* *i.e.*, the number of elements of *A_l_* and *A_l_*. In our case *card* (*A_l_*) is equal to 6, since we have six attributes. According to the definition in Equation (9), *d_i_* (*A_l_*, *A_i_*) = 1 if the attributes are equals (maximum similarity), otherwise if they are completely different *d_i_* (*A_l_*, *A_i_*) = 5/6 (minimum similarity). This value is obtained by assuming that if one attribute is null and the other the unity the absolute difference value between both is the unity and because ∑*_j_w_j_* = 1, this results in that minimum. Finally, the limits for minimum/maximum similarity are obtained by mapping linearly the above limits to range as follows: *d_i_* (*A_l_*, *A_i_*) ⊂ [0, 1].

The original similarity measure in [26,27] does not include the weight *w_j_*, we have included this weight because of the relative importance of each attribute, which means that each attribute contributes in a different fashion to the matching. In Section 4.2 we provide details about its computation. This makes a contribution of this work because it favors the correspondences.

As mentioned before, in this paper we use the following six attributes for describing each pixel (feature): (*a*) correlation; (*b*) texture; (*c*) colour; (*d*) gradient magnitude; (*e*) gradient direction and (*f*) Laplacian. Both first ones are catalogued as area-based, computed on a 3 × 3 neighbourhood around each pixel through the correlation coefficient [10] and standard deviation [6] respectively. The four remaining ones are considered as feature-based [11]. The colour involves the three red-green-blue spectral components (R,G,B) and the absolute value in the Equation (9) is extended as: |*A_lj_* − *A_ij_*| = ∑*_H_*|*H_lj_* − *H_ij_*|, H = R,G,B.

Gradient (magnitude and direction) and Laplacian are computed by applying the first (Sobel’s operator) and second derivatives [6], over the intensity image after its transformation from the RGB plane to the HSI (hue, saturation, intensity) one.

At this stage we have available the similarities between the pixel *l* in the left image and each pixel *i* in the list of *m* candidates. We must make a decision about the best match, which implies the mapping of the uniqueness constraint. The decision is made based on the following rule: *i* is the best match of *l* if *d_i_*(*A_l_*, *A_i_*) > *d_i_* (*A_l_*, *A_j_*), *i*, *j* = 1,…,*n; i* ≠ *j*.

### 3.3. Disparity Map Computation

Taking as reference the left image of the stereo pair, for each pixel *l* ≡(*x_l_*, *y_l_*) in this image we have its corresponding match in the right one *i* ≡(*x_i_*, *y_i_*). Therefore, we know their corresponding locations in Cartesian coordinates, which are transformed to polar coordinates considering the centre of the image as the origin of the polar reference system; so both pixels *l* and *i* have polar angles *θ_l_* and *θ_i_*, respectively. We build a map with the same locations that the original left image, *i.e.*, *q* = *M* × *N* (*M* rows, *N* columns), where each location represents a pixel. Given the pixel location *l* ≡(*x_l_*, *y_l_*) we load it with the following value Δ*θ_l_* = |*θ_l_* − *θ_i_|* which represents the disparity value for the pixel *l*, once it has been matched with its best candidate *i*. This process is carried out for all locations corresponding to unclassified pixels during the segmentation process. We assign a null disparity value for those locations corresponding to pixels classified as belonging to sky, grass or leaves. The values in the disparity map range in the interval [0, *θ*_max_], where *θ*_max_ is fixed to 6.0 in our approach because it is the maximum disparity value observed in all available stereo images. This is the initial disparity map which is used as input for the HNN approach.

### 3.4. Smoothness: Hopfield Neural Networks (HNN)

Once the disparity map is obtained according to the above process, we try its improvement based on the HNN paradigm. In Sections 3.4.1 and 3.4.2 we give details about the topology of a HNN and its working process. In Sections 3.4.3 and 3.4.4 we apply this paradigm for improving the incoming disparity map by applying the smoothness constraint.

#### 3.4.1. Topology and Basic Concepts

An important issue addressed in neural computation for image applications is referred to how sensory elements in a scene perceive the objects, *i.e.*, how the scene analysis problem is addressed. To deal with real-world scenes some criterion for grouping elements in the scene is required. In the work of [28] a list of major grouping principles is exhaustively studied. They are inspired in the Gestalt’s principles [29]. In our approach we apply the following three principles: *proximity*, labelled pixels that lie close in space tend to group; *similarity*, labelled pixels with similar values tend to group; *connectedness*, labelled pixels that lie inside the same connected region tend to group. These principles allow defining a spatial neighbourhood. Now the problem is to build some structure that can cope with the above. Several approaches can be used; we have chosen the HNN because it is an optimization one based on energy minimization, *i.e.*, the convergence can be controlled by the energy. In HNN the above principles can be applied by considering the influences exerted by the nodes *k* in a neighbourhood over a node *i* and mapped as consistencies, from the data and the contextual information, as explained later.

From the disparity map available at this moment, we build a network of nodes, where the topology of this network is established by the spatial distribution of the disparity map. Each node in the network is located at the same position that the elements in the map, *i.e.*, at the same position that the corresponding pixel in the left image with the associated disparity value. Hence, the number of nodes in the network is *q* = *M* × *N*. The node *i* in the network is initialized with the disparity value obtained from the disparity map at the same location, *i.e.*, Δ*θ_i_*, but instead of using the range [0, *θ*_max_] we map linearly the disparity values for ranging in [−1, +1]; for simplicity Δ*θ_i_* is renamed as *D_i_*.

The network states (activation levels) are the normalized disparity values associated to the nodes. Through the HNN these network states are reinforced or punished iteratively based on the influences exerted by their neighbours. The goal is to smooth the disparity map based on more stable state values.

#### 3.4.2. A Review on the HNN

The HNN paradigm initially proposed by Hopfield and Tank [30,31] has been widely used for solving optimization problems. This implies fixing two characteristics [32]: its activation dynamics and an associated energy function which decreases as the network evolves.

The HNN is a recurrent network containing feedback paths from the outputs of the nodes back into their inputs so that the response of such a network is dynamic. This means that after applying a new input, the output is calculated and fed back to modify the input. The output is then recalculated, and the process is repeated again and again. Successive iterations produce smaller and smaller output changes, until eventually the outputs become constant, *i.e.*, at this moment the network achieves an acceptable stability.

The connection weights between the nodes in the network may be considered to form a matrix *T*. Although some studies carried out by [33] in Hopfield neural networks have been addressed for solving the problem of optimal asymmetric associative memories, we have found acceptable the classical approach studied in [34] and [35] where it is shown that a recurrent network is stable if the matrix is symmetrical with zeros on its diagonal, that is, if *T_ik_* *= T_ki_* for all *i* and *k* and *T_ii_* *=* 0 for all neurons *i*. To illustrate the Hopfield networks in more detail, consider the special case of a Hopfield network with a symmetric matrix. The input to the *i^th^* node comes from two sources: external inputs and inputs from the other nodes. The total input *u_i_* to node *i* is then:

(10) $$u_{i}(t)=\sum_{k\neq i}T_{\text{ik}}D_{k}(t)+U_{i}(t)$$

where the *D_k_*(*t*) value represents the output of the *k^th^* node at the iteration *t; T_ik_* is the weight of the connection between nodes *i* and *k*; and *U_i_* represents an external input bias value which is used to set the general level of excitability of the network. There are two kinds of Hopfield networks [32,36] namely, (1) the analog ones in which the states of the neurons are allowed to vary continuously in an interval, such as [−1, +1] and; (2) the discrete ones in which these states are restricted to the binary values −1 and +1. The drawback of these binary networks is that they oscillate between different binary states, and settle down into one of many locally stable states. Hopfield has shown that analog networks perform better since they have the ability to smooth the surface of the energy function which prevents the system from being stucked in minor local minima [30,31].

For analog Hopfield networks the total input into a node is converted into an output value by a sigmoid monotonic activation function instead of the thresholding operation for discrete Hopfield networks [35]. The dynamic of a node is defined by:

(11) $$\frac{du_{i}}{\text{dt}}=-\frac{u_{i}}{R_{i}}+\sum_{k\neq i}T_{\text{ik}}D_{k}(t)+U_{i}\;\;\;\text{where}\;\;\;D_{k}(t)=g\left(u_{k}\right)\;\;\;\;\;\;\forall k$$

where *g*(*u_i_*) is the sigmoid activation function, and *R_i_* is a time constant which can be set to 1 for simplicity [36,37]. We have chosen the sigmoid activation function to be the hyperbolic tangent function [36], *g*(*u_k_*) = tanh(*u_k_/β*). This function is differentiable, smooth and monotonic, *i.e.*, contributes to the network stability [35]. A detailed discussion about the settings of the time step *dt* and gain *β*^−1^ can be found in [32]. As *dt* increases, the probability that the energy falls into a local minimum also increases. According to some experiments carried out in [32] where this parameter has been set to values in the range 1 to 10^−2^, the best performance is achieved with the minimum value (*i.e.*, 10^−2^), hence we have fixed it to 10^−3^ which is an order of magnitude less than the experimented in [32]. The way to avoid that a continuous network cannot find a solution due to the existence of local minimum and makes the network converge up to a solution state is to decrease *β* along the simulation, theoretically until *β* = 0. This strategy reminds a simulated annealing process starting from high enough *β*, then the network evolves until a stable state (which is not a solution) is reached, then *β* is decreased and the network evolves again up to a new stable state, and so on; the process ends when *β* becomes zero and at this moment, the stable state reached should be a global minimum. According to the results reported in [38] and [39], we have tested the following scheduling strategy *β*(t) = *β*_0_/log(t + 1) where *t* is the iteration number. We have computed *β_0_* as follows [40]: (1) we select four pairs of images, where the nodes have been initialized; now we compute the initial energy; (2) we choose an initial *β*, that permits about 80% of all transitions to be accepted (*i.e.*, transitions that decrease the energy function), and this value is changed until this percentage is achieved; (3) we compute the *M* transitions Δ*E_i_* and we look for a value for *β* for which $\frac{1}{M}\Sigma_{k=1}^{M}\text{exp}(-\Delta E_{k}/\beta )=0.8$, after rejecting the higher order terms of the Taylor expansion of the exponential, *β*= 5 〈Δ*E_k_*〉, where 〈·〉 is the mean value. In our experiments, we have obtained 〈Δ*E_k_*〉 = 0.87, giving *β*_0_ = 4.35. In the work of [39] a simulated annealing scheduling is used with *β*_0_ = 2, *i.e.*, with the same order of magnitude. Taking into account that *β*(*t*) = 0, *t* → +∞ and considering *t* = 10^10^ we obtain *β* = 0.43, *i.e.*, *β*^−1^ = 2.30. In our image classification approach, we have carried out different experiments by applying the above scheduling and also assuming fix the gain without apparent improvement in the final results. Hence we set the gain to 2.30 during the full process.

The model provided in Equation (11) is the classical Hopfield circuit [30,31,41] which follows from the Cohen-Grossberg dynamical systems [34]. In [41] the global stability of these systems is proven under the positivity assumption *dg*/*dt* > 0 and considering that the coefficient in the left term of Equation (11) is also positive. Because *g* is the hyperbolic tangent function the first condition is true. Additionally, towards the global stability also contributes that the bias *U_i_* varies slowly. In our design this is also true according to the discussion in Section 3.4.3(d). The stability of the Hopfield neural network has also been studied under different perspectives in [35] or [42]. Hence, it belongs to the important class of feedback neural networks models that are globally stable. The quantity describing the state of the network called energy, is defined as follows:

(12) $$E(t)=-\frac{1}{2}\sum_{i}\sum_{k\neq i}T_{\text{ik}}D_{i}(t)D_{k}(t)-\sum_{i}U_{i}D_{i}(t)+\beta \sum_{i}\int_{0}^{D_{i}}g^{-1}\left(D\right)dD$$

According to the results reported in [32], the integral term in (12) is bounded by *β*ln 2 ≈ 0.19 when *D_i_*(*t*) is +1 or −1 and is null when *D_i_*(*t*) is zero. In our experiments, we have verified that this term does not contribute to the network stability and only the energy is increased in a very little quantity with respect to the other two terms in Equation (12), hence for simplicity we have removed it from the Equation (12).

The continuous Hopfield model described by the system of nonlinear first-order differential Equation (11) represents a trajectory in phase space, which seeks out the minima of the energy function in (12).

#### 3.4.3. Mapping Consistencies and Information

##### (a) Consistency from the Data

During the optimization process the initial states *D_i_*(*t*) are modified trying to achieve the network stabilization. Now, the goal is to map the data consistency between nodes *i* and *k* into the *consistency* coefficient *w_ik_*(*t*), at each iteration *t*. Given the node *i* we consider its *m*-connected neighborhood $N_{i}^{m}$, under the grouping criterion established by the proximity and connectedness principles according to [28]; *m* could be 4, 8, 24, 48 or any other value taking into account only horizontal, vertical or diagonal directions. A typical value is 8, corresponding to a central pixel and its 8 neighbors.

For each node *i*, only consistencies can be established between nodes *k*, where $k\in N_{i}^{m}$ and *i* ≠ *k* otherwise if $k\notin N_{i}^{m}$ it is assumed that there is not consistency between nodes *i* and *k*. This is justified under the hypothesis that only local relations can be established between pixels with similar disparities. Two nodes *i*, *k* where $k\in N_{i}^{m}$ are said consistent if they have similar data information, *i.e.*, similar disparities. Otherwise they should be inconsistent. The data consistency between the nodes *i*, *k* is mapped into the consistency coefficient as follows:

(13) $$w_{\text{ik}}\;\left(t\right)=\{\begin{array}{lll} 1-\left|D_{i}\left(t\right)-D_{k}\left(t\right)\right| & k\in {\text{N}}_{i}^{m}, & i\neq k\\ 0 & k\notin {\text{N}}_{i}^{m}, & i=k \end{array}$$

From (13) we can see that *w_ik_*(*t*) ranges in [−1, +1]. The influence exerted by the node *k* over the node *i* will be positive (reward) or negative (punishment). Hence, a positive data consistency will contribute towards the network stability.

##### (b) Consistency from the Contextual Information

In some existing works dealing with images, such as in [38], the inter-pixel dependence is described by defining a kind of consistency which is achieved under the consideration of contextual information. We make use of this concept and apply it to our HNN approach. Given the node *i* at the pixel location (*x*,*y*) with state value *D_i_* and a set of nodes $k\in N_{i}^{m}$ with state values *D_k_*, a measurement of contextual consistency between the node *i* and its *k* neighbors can be expressed as:

(14) $$E_{i}(t)=\sum_{k\in N_{i}^{m}}D_{i}(t)D_{k}(t)$$

This term represents an inter-state relation between the nodes in the network. It also represents a kind of external influence exerted by the nodes *k* over the node *i*. As *D_i_*(*t*) and *D_k_*(*t*) range in [−1, +1], given *D_i_*(*t*) the term *E_i_*(*t*) will be maximum when the *D_k_*(*t*) values are close to *D_i_*(*t*). Indeed, assuming that under the 8-neighbourhood *D_i_*(*t*) and *D_k_*(*t*) take simultaneously values of +1 or −1, *E_i_*(*t*) = 8, *i.e.*, reaches its maximum value. On the contrary, if *D_i_*(*t*) = +1 and all *D_k_*(*t*) = −1 or *D_i_*(*t*) = −1 and all *D_k_*(*t*) = +1, *E_i_*(*t*) = −8, *i.e.*, its minimum value. It is worth noting that Equation (14) can be regarded as an implementation of the Gibbs potential in a neighborhood under the Markov Random Fields framework [38].

Once data and contextual consistencies are specified, we search for an energy function such that the energy is low when both consistencies are high and vice versa. This energy is expressed as:

(15) $$\begin{array}{l} E_{C}\;(t)=-\frac{A}{2}\sum_{i}\sum_{k\in N_{i}^{m}}\left\{\;{\left[\text{sgn}\;\left(w_{\text{ik}}\;(t)\right)\right]}^{v+1}w_{\text{ik}}\;(t)-\delta_{\text{ik}}\right\}D_{i}\;(t)D_{k}\;(t)\;\\ \text{where}\;\;\;\;\text{sgn}\;\left(w_{\text{ik}}\;(t)\right)=\{\begin{array}{cc} -1 & w_{\text{ik}}\;(t)\leq 0\;\\ +1 & w_{\text{ik}}\;(t)>0\; \end{array}\;\;\;\;\;\text{and}\;\;\;\;\;\delta_{\text{ik}}=\{\begin{array}{ccc} 1 & \text{if} & i=k\\ 0 & \text{if} & i\neq k \end{array} \end{array}$$

where *A* is a positive constant to be defined later, sgn is the *signum function* and *v* is the number of is negative values in the set *C* ≡{*w_ik_*(*t*), *D_i_*(*t*), *D_k_*(*t*)}, *i.e.*, given *S* ≡ {*s* ∈ *C* / *s <* 0} ⊆ *C*, *v* = card (*S*); *δ_ik_* is introduced to cancel the self contribution of the node *i* because it is considered later under the self-data information.

Table 1 shows the behavior of the energy term *E_C_*(*t*) against data and contextual consistencies. As one can see, the energy decreases as the data and the states are both simultaneously consistent (rows 1 and 4 in the left part of the Table 1); otherwise under any inconsistency the energy increases. We have considered that data inconsistencies have higher priority than contextual ones; so under this criterion if *w_ik_*(*t*) < 0 then the energy increases.

##### (c) Self-Data Information

We have analyzed the inter-relations between nodes in a given neighborhood, based on data and contextual consistencies. This implies that the state for each node evolves according to the information provided by the majority in the neighborhood, ignoring its own information. This may lead to an incorrect state for the node under consideration. To overcome this drawback we assume that each node must contribute to the evolution of its own state through the self-data information. The self-data information is modeled as a kind of self-consistency based on the hypothesis that a node in the network with a disparity value (state) *D_i_*(*t*) its updating must be governed by this value. This is mapped as follows:

(16) $$E_{B}\;(t)=-B\sum_{i}D_{i}(t)D_{i}(t)$$

The constant *B* is a positive number to be defined later. So, independently if *D_i_* is positive or negative, the product *D_i_*(*t*)*D_i_*(*t*) is always positive and the term *E_B_*(*t*), at each iteration is minimum, as expected.

##### (d) Derivation of the Connection Weights and the External Inputs for the HNN

Assuming data and contextual consistencies, Equation (15), and self-data information, Equation (16), we derive the energy function of the Equation (17), which is to be minimized by optimization under the HNN framework:

(17) $$\begin{array}{l} E(t)=E_{C}\;(t)+E_{B}\;(t)=\\ -\frac{A}{2}\sum_{i}\sum_{k\in N_{i}^{m}}\left\{\;{\left[\text{sgn}\;\left(w_{\text{ik}}\;(t)\right)\right]}^{v+1}w_{\text{ik}}\;(t)-\delta_{\text{ik}}\right\}D_{i}(t)D_{k}(t)-B\sum_{i}D_{i}(t)D_{i}(t) \end{array}$$

By comparison of the expressions Equations (12) and (17) without the integral term in Equation (12), it is easy to derive the connection weights and the external input bias as follows:

(18) $$T_{\text{ik}}=A{\left[\text{sgn}\;\left(w_{\text{ik}}\;(t)\right)\right]}^{v+1}w_{\text{ik}}\;(t)-\delta_{\text{ik}};\;U_{i}\;(t)={\mathrm{BD}}_{i}(t)$$

According to the discussion in Section 3.4.2, to ensure the convergence to stable state [34], symmetrical inter-connection weights and no self-feedback are required, *i.e.*, we see that by setting A = B = 1 both conditions can easily be derived from (17). Also, the external input bias *U_i_*(*t*) must vary slowly to ensure the network stability. Because the network is loaded initially with the disparity map provided by the WFS approach, the network optimization process starts with a high degree of stability and these values change slowly. Additionally, the definition of the neighborhood establishes that only small numbers of neurons are interconnected. It is also well-known that this contributes to the stability [42].

The energy in Equation (17) represents a trade-off between the data and contextual information coming from the spatial environment surrounding the node *i* and also its self-data information. The constants *A* and *B* could be fixed so that they tune the influence of each term in Equation (17). We have carried out several experiments verifying that in our approach the above setting is appropriated.

The Equation (11) describes the time evolution of the network, the total input to the node *D_i_*(*t*) is computed by solving the Equation (11) with the Runge-Kutta method. Finally, the state *D_k_*(*t*) is also computed according to Equation (11). As we can see, the energy in Equation (17) is obtained by considering the state values and a kind of attractiveness derived from both, data and contextual consistencies. The derivation of an energy function with attractiveness between fixed points has been well-addressed in the work of [43] for discrete Hopfield memories preserving symmetrical weights and without self-feedback. Hence, we can assume that under the attractiveness of data and contextual consistencies, our analog Hopfield approach performs appropriately.

#### 3.4.4. Summary of the Smoothness Constraint Mapping

After mapping the energy function onto the Hopfield neural network, the filtering of the disparity map is achieved by letting the network evolve so that it reaches a stable state, *i.e*., when no change occurs in the states of its nodes during the updating procedure. The whole smoothness procedure can be summarized as follows:

1. *Initialization*: create a node *i* for each pixel location (*x*,*y*) from the left image; *t* = 0 (iteration number); load each node with the state value *D_i_*(*t*) defined in Section 3.4.1; compute *T_ij_*, *U_i_*(*t*) through Equation (18); *ɛ* = 0.01 (a constant to accelerate the convergence); *t_max_* = 20 (maximum number of iterations allowed); set the constant values as follows: *R_i_* = 1; *β* = 0.43; *dt* = 10^−3^. Define *nc* as the number of nodes that change their state values at each iteration.
2. *HNN process*: set *t* = *t* + 1 and *nc* = 0; *for* each node *i* compute *u_i_*(*t*) using the Runge-Kutta method and update *D_i_*(*t*), both according to Equation (11) and *if* |*D_i_*(*t*)−*D_i_*(*t* − 1)|> ɛ *then nc* = *nc +* 1; when all nodes *i* have been updated, *if nc* ≠ 0 and *t* <*t*_max_ *then* go to step 2 (new iteration), *else* stop.
3. *Outputs: D_i_*(*t*) updated for each node (disparity values for each pixel location).

## 4. Results

The system geometry is based on the scheme of the Figure 3, with a baseline of 1 meter. The camera is equipped with a Nikon FC-E8 fisheye lens, with an angle of 183°. The valid colour images in the circle contain 6,586,205 pixels.

The tests have been carried out with twenty pairs of stereo images. The total number of pairs of pixels extracted from these images is 131,724,100. This number of pairs of pixels is representative of the forest environment where the measurement device works, *i.e.*, Scots pine forest (*Pinus sylvestris* L.). We use four pairs of them for the training involved in the FC and PB approaches (Section 2.2.1) and also for computing the relevance of each criterion from which the fuzzy weights, involved in the WFS approach (Section 3.2), can be obtained.

At a second stage, for the remainder sixteen stereo pairs we obtain the initial disparity map for each stereo pair by applying the WFS approach pixel by pixel (Section 3.2). Then, each initial disparity map is smoothed through the HNN method (Section 3.4).

The tests consist on the computation of the errors obtained in the disparity maps. For such purpose we have available the ground truth disparity maps for the trunks of each stereo pair, provided by the end users. Thus, for each pixel in a trunk we know its correct disparity value according to this expert knowledge; which allows us to compute the percentage of error. For each one of the sixteen pairs of stereo images used for testing, we compute the disparity error for the pixels belonging to the trunks and then average these errors among the sixteen pairs of stereo images. Only the trunks located in an area of 25 m^2^ around the stereo vision system are to be tested, because for the trunks out of this area the volume of wood cannot be obtained with the required precision.

In the remainder of this section we give details, in Section 4.1, about the results obtained by the segmentation process described in Section 2, including the training and decision phases. In Section 4.2 we display the results obtained for the relevance of each criterion, to be used in the WFS. In Section 4.3 we display the performance of the WFS and the HNN. They are compared against the results obtained by applying each criterion separately, also by applying only the WFS strategy, and against the combined decision making strategies of Yager [44], the Choquet Fuzzy Integral (CFI) [45], the Sugeno Fuzzy Integral (SFI) and the Dempster-Shafer theory (DES) [46], and the Fuzzy Multicriteria Decision Making (FMCDM) [24].

### 4.1. Results for the Training Phase during the Image Segmentation Process

From the four pairs of stereo images available for this, we select manually the samples belonging two the sky and grass textures, obtaining a set of 2,560 samples, which are used for estimating the cluster centres involved in Equations (2) to (4). As one can see from the image in Figure 1, the grass texture displays several intensities values depending on if the pixels are in a shaded or a sunny area. Therefore, to avoid problems with the absolute values of the R,G,B spectral components, we normalize them to range in the interval [0,1] as follows: given a sample ***x*** = (*R*,*G*, *B*) it is normalized as ***x*** = (*R/U* ,*G/U* , *B/U*) with *U* = *R* + *G* + *B*. The cluster centres obtained by applying the training process in Section 2.2.1 through Equations (2) to (4) are displayed in Table 2.

Table 3 shows the most significant thresholds and parameters values involved in the pixel-based segmentation process, depending on whether they are related to the training or decision phase. Figure 2 displays the results obtained after segmentation for the image in Figure 1(a), where high contrasted areas are identified through the coefficient *Z*, Equation (1). Sky and grass textures are identified through the combined approach during the decision phase in Subsection 2.2.2. In summary, as one can see in Figure 2, the white pixels have been identified as belonging to one of the three textures out of interest, which are discarded during the later matching process, making it easier.

It is difficult to validate the results obtained by the segmentation process, but we have verified that without the segmentation process, the error for all matching strategies is increased a quantity that represents on average about 9–10 percentage points. In addition to this qualitative improvement it is easy to deduce the existence of a quantitative improvement by the fact that some pixels belonging to textures not excluded by the absence of segmentation, they are incorrectly matched with pixels belonging to the trunks, this does not occur when these textures are excluded, because they were not offered this possibility. This means that the segmentation is a fundamental process in our stereovision system.

### 4.2. Computing the Relevance for Each Criterion

Given the four available pairs of stereo images for this purpose, we selected manually a set of 800 pixels belonging to the trunks in the left images and their corresponding true matches in the right one, obviously also in the matched trunk, according to our expert knowledge. So, given a pixel *l* coming from a left image we apply the stereovision matching process described in Section 3 and search for a set of *m* potential matches according to each individual attribute identified by *j*, *i.e.*, the similarity between *l* and each *i* of the *m* candidates is computed according to the following expression for the attribute *j*:

(19) $$s_{j}\;(l,i)=1-\left|A_{\text{lj}}-A_{\text{ij}}\right|;\;i=1,..,m;\;j=a,b,c,d,e,f;$$

where *s_j_* (*l*,*i*)∈[0, 1], *s_j_* (*l*,*i*) = 1 if the difference between both attributes is null (maximum similarity), otherwise if they are very different, *s_j_*(*l*,*i*) = 0 (minimum similarity). Because we know the correct matches we are able to compute the averaged percentage of error for the set of 800 pixels based on each attribute and hence the relative relevance and the weights *w_j_* for the individual attributes. These percentages are finally: *p_a_* = 30 (correlation), *p_b_* = 12 (colour), *p_c_* = 16 (texture), *p_d_* = 10 (gradient magnitude), *p_e_* = 34 (gradient direction) and *p_f_* = 30 (Laplacian). So, each weight is computed as *w_h_* = *p_h_*/∑*_k_p_k_*, *h, k* = *a,b,c,d,e,f*. The weights associated with every attribute are respectively *w* = 10^−3^(227,90,122,76,258,227). As one can see, the most relevant attribute is the gradient magnitude.

### 4.3. WFS and HNN Performances

**Figure 4.**
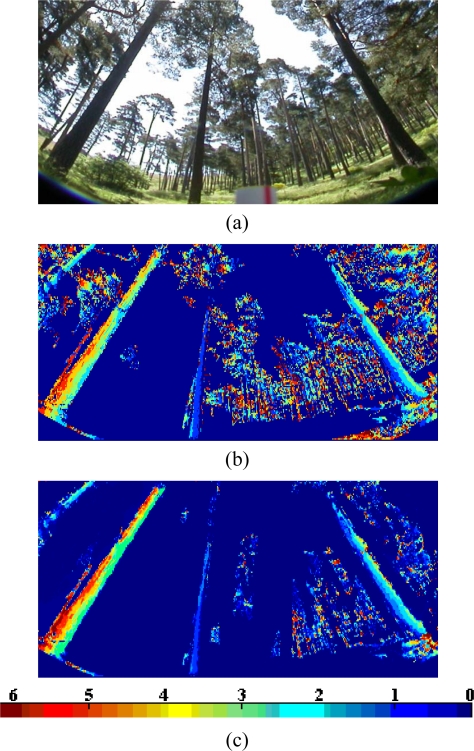
**(a)** Expanded area corresponding to the signed area in the image of Figure 1(a); **(b)** disparity map obtained by the WFS approach; **(c)** disparity map obtained by the HNN approach.

Given a stereo pair of the sixteen used for testing, for each pixel we obtain its disparity as follows. For facility, we reproduce in Figure 4(a) the expanded area in Figure 1(b).

**Figure 5.**
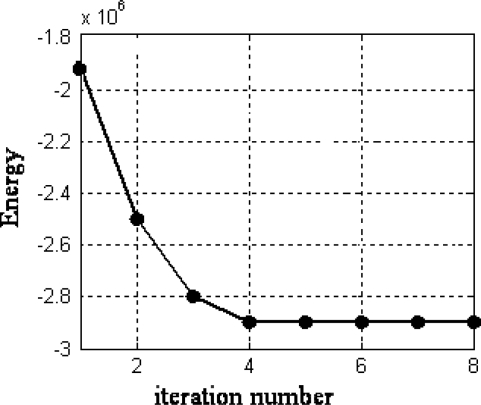
Energy variation against the number of iterations during the HNN optimization process.

Considering the six attributes separately, used as criteria in the WFS, and applying a maximum similarity criterion according to Equation (9) among the *m* candidates, we obtain a disparity map for each attribute. By applying the WFS approach based on maximum similarity, we obtain the initial disparity map displayed in Figure 4(b) for the area in Figure 4(a). This initial map is filtered (smoothed) through the HNN procedure. After four iterations of the HNN we obtain the disparity map displayed in Figure 4(c). The colour bar shows the disparity level values according to the colour for each disparity map. We have verified that more iterations do not improve the map. This is explained because as displayed in Figure 5, the energy reaches a stable value at the iteration 4 and then remains stable for the other iterations. This is the general behaviour for the remainder stereo images. The average number of iterations for the sixteen stereo pairs is 3.8.

As one can see by observing the disparity map in Figure 4(c), many isolated disparity values out and inside the trunks in Figure 4(b) have been changed towards the values given by their neighbours. This leads to the desired smoothing in both the trunks and outside them. Another important observation comes from the main trunk in the left part of the expanded area; indeed, in the initial map, Figure 4(b), the disparity values range from 1.5 to 5.5, but in the filtered map, Figure 4(c), the low level values have been removed, now the disparities range from 3.5 to 5.5. Although there are still several disparity levels, this is correct because the trunk is very thick and it is placed near the sensor. This assertion is verified by the expert human criterion.

Table 4 displays the averaged percentage of errors and standard deviations based on the similarity for the six attributes when used separately, identified under the follows columns: (*s_a_*, *s_b_*, *s_c_*, *s_d_*, *s_e_*, *s_f_*). The averaged percentage of error obtained with the WFS and the HNN approaches are also displayed.

Because the WFS approach is a combined decision making strategy, for comparative purposes we have tested the performance of our methods (WFS and HNN) against the combined decision making approach proposed by Yager [44] based on fuzzy sets aggregation. The combination is made two to two similarity measures as defined in Equation (19) according to the following expression:

(20) $$S\left(l,i\right)=1-\text{min}\left\{1,\;{\left({\left(1-s_{h}(l,i)\right)}^{p}+{\left(1-s_{k}(l,i)\right)}^{p}\right)}^{\frac{1}{p}}\right\},\;p\geq 1$$

where *h* and *k* denote two similarity measures. Then, by applying the associative property of this aggregation operator we compute a final support for the six similarity values. The parameter *p* is estimated from the four stereo pairs used for training and computing the relevance of each attribute. Indeed, we vary *p* from 1 to 4, which is the range generally used, and compute the percentage of error, obtaining the best results with *p* set to 2.0. The averaged percentage of error obtained trough the Yager method (YAG) are also displayed in Table 4.

Also for comparative purposes, we have tested the performance of our proposed methods against the combined decision making approaches successfully used in previous works in the same forest environment. Concretely, the combination in each method is: in CFI as we explain in [45], in the SFI and DES methods as in [46], and in the FMCDM method as in [24]. The averaged percentage of error and standard deviations obtained trough these three methods are also displayed in Table 4.

From results in Table 4 one can see that the strategies that combine the simple attributes, YAG, CFI, SFI, DES, FMCDM and WFS, outperform the individual similarity based approaches. This means that the combination of similarities between attributes improve the results obtained by using similarities separately. These conclusions have been already obtained in our previous works [24,45,46].

The best individual results, according to the six attributes, are obtained through the similarities provided by the gradient magnitude attribute (*s_d_*). This implies that it is the most relevant attribute. This agrees with its relevance obtained previously in Section 4.2, as it has turned out to be the most relevant attribute.

The combined strategies that show better results they are FMCDM and WFS. Both of them obtain similar results in terms of percentage and with the lower standard deviation, *i.e.*, with less dispersion of the values from statistical point of view. The main advantage for using WFS is its simplicity with respect FMCDM and therefore it implies a lower computation cost.

Nevertheless, the main relevant results are obtained by the proposed HNN approach in terms of less percentage of error. This together with the qualitative improvement provided by this approach, as explained above, allow us to conclude that it is a suitable method for computing the disparity map in this kind of images.

We have verified that without the segmentation process the error for all strategies is increased about a quantity that represents on average about 9–10 percentage points. This means that the segmentation process is very important.

**Figure 6.**
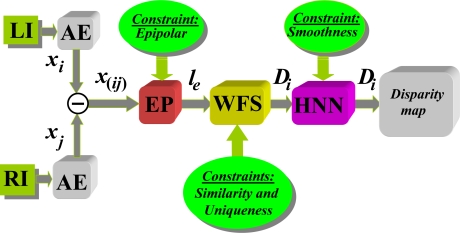
Proposed stereoscopic matching process based on pixels.

To sum up the study, the strategy shown in diagram in Figure 6 is proposed based on the results obtained with different methods. The diagram first shows the capture of the two images involved in the LI (Left Image) and RI (Right Image) process with the geometric configuration system described in Section 3.1, resulting in the implementation of epipolar constraint. Then the segmentation process is applied for excluding textures, after which it is defined the properties (attributes) of the pixels that are used as features for correspondence. This process is applied in parallel to the two stereoscopic images that in Figure 6 has been identified as AE. The application of the epipolar constraint through the relevant process is identified in the Figure 6 as EP where *l_e_* is the epipolar line obtained from Equations (5) to (8). The similarity constraint provides values of similarity between pixels in one picture to another so that by the application of the uniqueness constraint, with details specified previously, it is decided on the best possible candidates. In both cases under the WFS approach because of the best results we obtained. Following the application of this method is obtained an initial disparity map, which is refined later through the HNN process by applying the smoothness constraint, with which we obtain the final disparity map.

## 5. Conclusions and Future Work

In this paper we have proposed a new strategy for obtaining a disparity map from hemispherical stereo images captured with fish-eye lenses. A first segmentation process identifies three types of textures, where the pixels classified as belonging to one of them are not matched. This improves the final results. The stereovision matching process is based on the application of four stereovision matching constraints.

An initial disparity map is obtained by applying three of them (*epipolar*, *similarity* and *uniqueness*). For each pixel in the left image, a list of possible candidates in the right one is obtained for determining its correspondence. This is carried out through the WFS approach, which is a decision making strategy based on a weighted fuzzy distance.

The initial disparity map is improved by applying the *smoothness* stereovision matching constraint, inspired on the Gestalt’s principles. This is carried out through the network built under the HNN paradigm, which can cope with the relations established between a pixel and its neighbours.

The proposed combined WFS strategy outperforms the methods that use similarities separately and also the combined decision making methods: YAG, CFI, SFI and DES. FMCDM and WFS obtain similar results although WFS is simpler than FMCDM and therefore it implies a lower computation cost. The HNN outperforms the WFS, thanks to the optimization process. This means that it is a suitable strategy for filtering disparity maps.

Based on this, some optimization approaches could be used, such as simulated annealing, where also the smoothness constraint and the Gestalt principles could be applied under an energy minimization based process.

This paper deals only with the stereovision matching problem in the specific forest environment studied. The method proposed can be applied for similar forest environments where pixels are the key features to be matched. Applications using this sensor are based on identical geometry and image projection, although the matching strategy could be completely different. This occurs in [47] where the matching strategy, based on region segmentation, is applied in Rebollo oak forests where the images are very different and captured under different illumination conditions.

Because the works based on this sensor are relatively recent, we have no reliable records for forest inventories. The next work following this study is the computation of distances to the trees for obtaining volumes and other variables of interest. Moreover, the matching strategy, proposed in this paper, could be considerably improved by considering previous and validated results obtained in the past, such as distances to the trees. This is because the trees, although growing, are fixed at a specific location with respect the sensor and a great volume of data are stationary, allowing to guide the matching process, where some ambiguities and false correspondences can be solved based on this information.

## Figures and Tables

**Table 1.** Behavior of the energy term against data and contextual consistencies.

*w_ik_*(*t*)	*D_i_*(*t*)	*D_k_*(*t*)	*E_C_*(*t*)	*w_ik_*(*t*)	*D_i_*(*t*)	*D_k_*(*t*)	*E_C_*(*t*)
+	+	+	−	−	+	+	+
+	+	−	+	−	+	−	+
+	−	+	+	−	−	+	+
+	−	−	−	−	−	−	+

**Table 2.** Cluster centres for the sky and grass textures.

	
	**Sky**	**Grass**
FC	***v***_1_ =(0.18,0.35,0.48)	***v***_2_ =(0.32,0.43,0.17)
PB	***v***_1_ =(0.16,0.32,0.52)	***v***_2_ =(0.31,0.48,0.14)

**Table 3.** Parameters and thresholds involved in the process of pixel-based segmentation.

**Phase**	**Parameters/thresholds**
Training	*e* = 2.1
	*ɛ* = 0.1
	*k_max_* = 20

Decision	*T*_1_ = 0.8
	*T*_2_ = 0.8

**Table 4.** Averaged percentage of errors and standard deviations obtained through maximum similarity criteria for each attribute separately and also for the WFS decision making approach and the HNN paradigm against the combined decision making strategies.

Averaged percentage of error and standard deviations			
**Category**	**Criteria/methods**	**%**	*σ*
Attributes	s*_a_* (correlation)	30.1	2.9
	s*_b_* (color)	16.2	1.3
	s*_c_* (texture)	18.1	1.7
	s*_d_* (gradient magnitude)	14.3	1.1
	s*_e_* (gradient direction)	35.2	3.6
	s*_f_* (Laplacian)	32.1	3.1

Decision making strategies	YAG	13.3	1.9
	CFI	11.2	1.3
	SFI	11.2	1.3
	DES	11.2	1.6
	FMCDM	9.3	0.9
	WFS	9.3	0.8

Filtering	HNN	**6.3**	**0.8**
